# Molecules New Aims and Scope

**DOI:** 10.3390/molecules22091510

**Published:** 2017-09-20

**Authors:** Derek J. McPhee

**Affiliations:** Editor-in-Chief, MDPI AG, St. Alban-Anlage 66, 4052 Basel, Switzerland; mcphee@mdpi.com

As we approach the end of our 22nd year as the pioneering and preeminent Open Access journal in the field of organic chemistry and natural products, time has come to formally announce what has been the de facto reality of the journal for the past few years, the expansion of the range of topics we cover.

Originally dedicated exclusively to organic synthesis and natural products chemistry, the evolution of these fields into multidisciplinary areas where the lines between chemistry, biology and materials are blurred, has led us to publish over the years increasing numbers of papers that did not fit the classical definition on the journal’s masthead. 

Moreover, we have not been alone in this evolution, and as several other MDPI journals have also refocused their own fields of coverage over the years, we felt that this left some interdisciplinary topics without a suitable forum. To remedy all this we are now formally expanding the scope of *Molecules* (ISSN 1420-3049, CODEN: MOLEFW), which henceforth will include (but will not be limited to):

- Organic chemistry
- Medicinal chemistry
- Natural products
- Inorganic chemistry
- Physical chemistry
- Materials science
- Nanoscience
- Catalysis
- Chemical biology
- Analytical chemistry
- Supramolecular chemistry
- Theoretical chemistry
- Green chemistry
- Photochemistry

As always, we encourage any authors who feel that their work does not fit into one of these boxes, to think of us as a venue for publishing their results. This refocus in no way changes our commitment to provide rigorous peer review and enable rapid publication of cutting-edge research to educate and inspire the scientific community worldwide. As our main objective is the full diffusion of experimental details and theoretical results, there will still be no restriction on the length of the papers, we will continue to insist on the publication of all experimental results and to encourage collaboration, we will continue to alert readers of the availability of samples and encourage the deposit and diffusion of chemical samples through the international non-profit organization Molecular Diversity Preservation International (MDPI). On behalf of the publisher and our Editorial Staff, I look forward to your thoughts and comments as we expand our Editorial Board to bring on experts in these new fields and especially, to receiving and publishing your papers.

